# Aqua­(2,2′-bipyridine-κ^2^ *N*,*N*′)[2-(3-thien­yl)malonato-κ^2^ *O*,*O*′]zinc(II) dihydrate

**DOI:** 10.1107/S1600536809047448

**Published:** 2009-11-18

**Authors:** Cai-Xia Meng, Xue-Gang Zheng, Feng Fu, Xiao-Ning Zhang, Peng Zhang

**Affiliations:** a Department of Chemistry and Chemical Engineering, Shaanxi Key Laboratory of Chemical Reaction Engineering, Yan’an University, Yan’an 716000, People’s Republic of China; b Lanzhou Institute of Biological Products, Lanzhou 730046, People’s Republic of China

## Abstract

In the crystal structure of the title compound, [Zn(C_7_H_4_O_4_S)(C_10_H_8_N_2_)(H_2_O)]·2H_2_O, the Zn^II^ ion assumes a trigonal–bipyramidal coordination geometry completed by two N atoms from a 2,2′-bipyridine ligand, two O atoms from a 2-(3-thien­yl)malonate anion and a water mol­ecule. The S atom of the 2-(3-thien­yl)malonate ligand is disordered over two sites with an occupancy ratio of 0.701 (5):0.299 (5). Inter­molecular O—H⋯O hydrogen bonding is present in the crystal structure.

## Related literature

For general background to organic heterocycles, see: Lin *et al.* (2008 ▶); Jin *et al.* (2001 ▶). For related thio­phene­malonate complexes, see: He *et al.* (2009 ▶); Murray *et al.* (2008 ▶); Huang *et al.* (2009 ▶); Lim *et al.* (2006 ▶). For hydrogen-bonded rings in polymeric complexes, see: Eppel & Bernstein (2009 ▶); Etter (1990 ▶); Nichol & Clegg (2009 ▶).
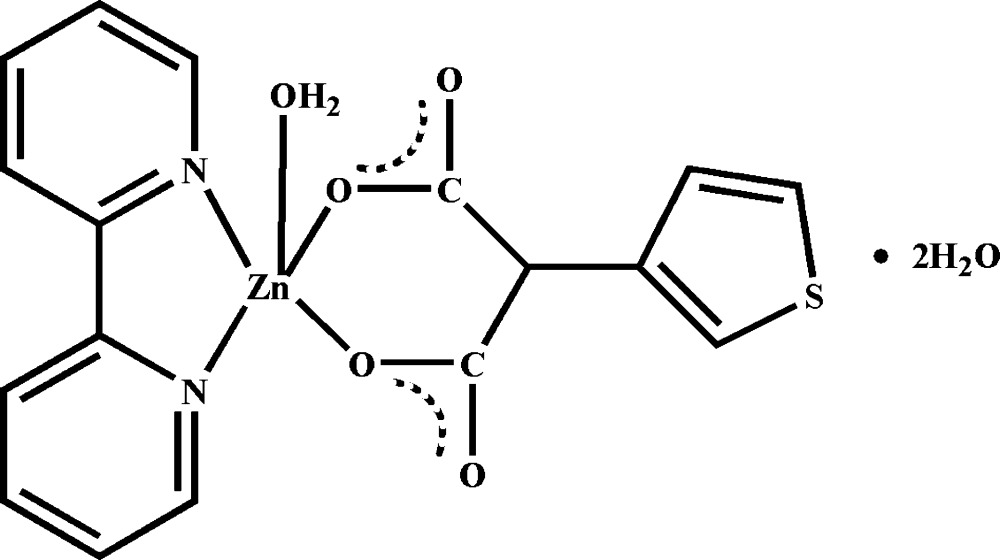


## Experimental

### 

#### Crystal data

[Zn(C_7_H_4_O_4_S)(C_10_H_8_N_2_)(H_2_O)]·2H_2_O
*M* *_r_* = 459.76
Orthorhombic, 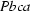

*a* = 15.9978 (10) Å
*b* = 14.5647 (9) Å
*c* = 16.5889 (10) Å
*V* = 3865.3 (4) Å^3^
*Z* = 8
Mo *K*α radiation
μ = 1.42 mm^−1^
*T* = 293 K
0.15 × 0.10 × 0.06 mm

#### Data collection

Bruker SMART CCD diffractometer
Absorption correction: multi-scan (*SADABS*; Sheldrick, 1996 ▶) *T*_min_ = 0.843, *T*_max_ = 0.918
23919 measured reflections
4752 independent reflections
3107 reflections with *I* > 2σ(*I*)
*R*_int_ = 0.045

#### Refinement

*R*[*F* ^2^ > 2σ(*F* ^2^)] = 0.035
*wR*(*F* ^2^) = 0.094
*S* = 1.01
4752 reflections
273 parameters
1 restraint
H-atom parameters constrained
Δρ_max_ = 0.29 e Å^−3^
Δρ_min_ = −0.39 e Å^−3^

### 

Data collection: *SMART* (Bruker, 1997 ▶); cell refinement: *SAINT* (Bruker, 1997 ▶); data reduction: *SAINT*; program(s) used to solve structure: *SHELXTL* (Sheldrick, 2008 ▶); program(s) used to refine structure: *SHELXTL*; molecular graphics: *SHELXTL*; software used to prepare material for publication: *SHELXTL*.

## Supplementary Material

Crystal structure: contains datablocks I, global. DOI: 10.1107/S1600536809047448/xu2654sup1.cif

Structure factors: contains datablocks I. DOI: 10.1107/S1600536809047448/xu2654Isup2.hkl

Additional supplementary materials:  crystallographic information; 3D view; checkCIF report

## Figures and Tables

**Table 1:** Selected bond lengths (Å)

Zn1—O1	2.0001 (15)
Zn1—O3	2.0224 (15)
Zn1—O5	1.9596 (15)
Zn1—N1	2.1123 (18)
Zn1—N2	2.100 (2)

**Table 2:** Hydrogen-bond geometry (Å, °)

*D*—H⋯*A*	*D*—H	H⋯*A*	*D*⋯*A*	*D*—H⋯*A*
O5—H18⋯O7^i^	0.85	1.74	2.568 (2)	165
O5—H19⋯O4^ii^	0.85	1.83	2.618 (2)	155
O6—H20⋯O2	0.85	1.94	2.784 (2)	172
O6—H21⋯O4^iii^	0.85	2.22	2.943 (2)	144
O6—H21⋯O3^iii^	0.85	2.45	3.238 (2)	154
O7—H22⋯O2	0.85	2.04	2.847 (3)	158
O7—H22⋯O1	0.85	2.47	3.165 (2)	139
O7—H23⋯O6^iv^	0.85	1.88	2.700 (3)	162

Symmetry codes: (i) 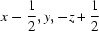; (ii) 
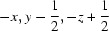
; (iii) 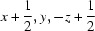; (iv) 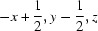.

**Fig. 1.**
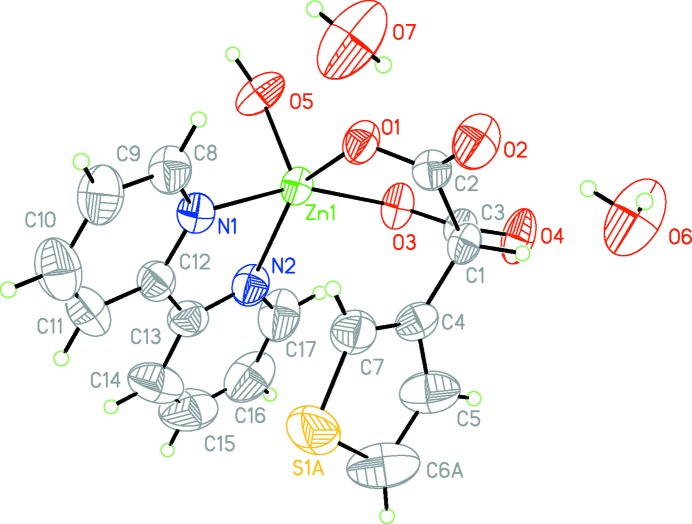
The structure of title complex,with the atom-numbering scheme for the asymmetric unit, showing displacementellipsoids at the 50% probability level.

## supplementary crystallographic information

### Comment

The nature of organic heterocycles are special enough to attract a lot of scientific researches (Lin *et al.*, 2008; Jin *et al.*, 2001). Because of the big radius of the S atom, its lone pair of electrons can be more easily delocalized with in the heterocycle, and the ligand exhibits good charge-transfer ability (He *et al.*, 2009). However, its reaction mechanism is rather complicated to study, a small quantity of literatures about thiophenemalonic are reported, with thiophenemalonic acid researched even less (Murray *et al.*, 2008; Huang *et al.*, 2009; Lim *et al.*, 2006). In this paper, we report the hydrothermal synthesis and structure of a new compound incorporating the thiophene-containing ligand3-thiophenecarboxylate with two kinds of hydrogen bond rings (Nichol *et al.*, 2009; Etter, 1990; Eppel *et al.*, 2009).

The molecular structure of the title complex is shown in Fig.1. The coordination geometry around Zn^2+^ ion is five-coordinated, with two N atoms from 2,2'-bipyridine ligand (N1, N2), two O atom from 3-thiophenecarboxylate ligand (O1,O3), and a coordinated water(O5). The equatorial positions are occupied by N2, O1 and O5, while N1, O3 are in axial positions, so the local coordination environment of Zn(II) can be described as a distorted trigonal bipyramidal environment (Table 1). The S1 and C6 atoms of the 3-thiophenemalonate ligand are disordered over two sites with refined occupancies of 0.701 (5) and 0.299 (5). The units are interconnected by two kinds of O–H···O hydrogen bonds rings (*R*_3_^3 ^(12), *R*_4_^3^(12)) to form a two-dimensional supramolecular network, in which the lattice water molecule acts as both hydrogen-bond donor and acceptor (Table 2). These hydrogen bonds rings contributes to the additional stability of the structure.

### Experimental

A mixture of Zn(NO_3_)_2_.6H_2_O (0.030 g, 0.1 mmol), 2,2'-bipyridine (0.008 g, 0.05 mmol), 3-thiophenemalonic acid (0.018 g, 0.1 mmol), NaOH (0.008 g, 0.2 mmol) and distilled water(10 ml) was sealed in a 25 ml Teflon-lined stainless autoclave and heated at 413 K for 72 h under autogenous pressure. After cooled at room temperature over 48 h, colorless crystals of the title compound suitable for X-ray analysis were obtained from the reaction mixture by filtration.

### Refinement

All H atoms were positioned geometrically (C—H = 0.93 and O—H = 0.85 Å) and allowed to ride on their parent atoms, with *U*_iso_(H) = 1.2*U*_eq_(C) or 1.5*U*_eq_(O). The S1 and C6 atoms of the 3-thiophenemalonate ligand are disordered over two sites, occupancies were refined to 0.701 (5):0.299 (5).

### Crystal data

**Table:**

[Zn(C_7_H_4_O_4_S)(C_10_H_8_N_2_)(H_2_O)]·2H_2_O	*F*(000) = 1888
*M**_r_* = 459.76	*D*_x_ = 1.580 Mg m^−^^3^
Orthorhombic, *P**b**c**a*	Mo *K*α radiation, λ = 0.71073 Å
Hall symbol: -P 2ac 2ab	Cell parameters from 4757 reflections
*a* = 15.9978 (10) Å	θ = 2.3–28.3°
*b* = 14.5647 (9) Å	µ = 1.42 mm^−^^1^
*c* = 16.5889 (10) Å	*T* = 293 K
*V* = 3865.3 (4) Å^3^	Prism, colorless
*Z* = 8	0.15 × 0.10 × 0.06 mm

### Data collection

**Table:**

Bruker SMART CCD diffractometer	4752 independent reflections
Radiation source: fine-focus sealed tube	3107 reflections with *I* > 2σ(*I*)
graphite	*R*_int_ = 0.045
φ and ω scans	θ_max_ = 28.3°, θ_min_ = 2.3°
Absorption correction: multi-scan (*SADABS*; Sheldrick, 1996)	*h* = −20→21
*T*_min_ = 0.843, *T*_max_ = 0.918	*k* = −19→19
23919 measured reflections	*l* = −13→21

### Refinement

**Table:**

Refinement on *F*^2^	Secondary atom site location: difference Fourier map
Least-squares matrix: full	Hydrogen site location: inferred from neighbouring sites
*R*[*F*^2^ > 2σ(*F*^2^)] = 0.035	H-atom parameters constrained
*wR*(*F*^2^) = 0.094	*w* = 1/[σ^2^(*F*_o_^2^) + (0.0445*P*)^2^ + 0.5447*P*] where *P* = (*F*_o_^2^ + 2*F*_c_^2^)/3
*S* = 1.00	(Δ/σ)_max_ = 0.013
4752 reflections	Δρ_max_ = 0.29 e Å^−^^3^
273 parameters	Δρ_min_ = −0.39 e Å^−^^3^
1 restraint	Extinction correction: *SHELXTL* (Sheldrick, 2008), Fc^*^=kFc[1+0.001xFc^2^λ^3^/sin(2θ)]^-1/4^
Primary atom site location: structure-invariant direct methods	Extinction coefficient: 0.0056 (3)

### Special details

**Table:**

Geometry. All e.s.d.'s (except the e.s.d. in the dihedral angle between two l.s. planes) are estimated using the full covariance matrix. The cell e.s.d.'s are taken into account individually in the estimation of e.s.d.'s in distances, angles and torsion angles; correlations between e.s.d.'s in cell parameters are only used when they are defined by crystal symmetry. An approximate (isotropic) treatment of cell e.s.d.'s is used for estimating e.s.d.'s involving l.s. planes.
Refinement. Refinement of *F*^2^ against ALL reflections. The weighted *R*-factor *wR* and goodness of fit *S* are based on *F*^2^, conventional *R*-factors *R* are based on *F*, with *F* set to zero for negative *F*^2^. The threshold expression of *F*^2^ > σ(*F*^2^) is used only for calculating *R*-factors(gt) *etc*. and is not relevant to the choice of reflections for refinement. *R*-factors based on *F*^2^ are statistically about twice as large as those based on *F*, and *R*- factors based on ALL data will be even larger.

### Fractional atomic coordinates and isotropic or equivalent isotropic displacement parameters (Å^2^)

**Table:**

	*x*	*y*	*z*	*U*_iso_*/*U*_eq_	Occ. (<1)
Zn1	0.006504 (15)	0.702960 (16)	0.153476 (15)	0.03795 (11)	
S1A	0.1680 (2)	0.8323 (2)	−0.04031 (12)	0.0838 (10)	0.701 (5)
C6A	0.1110 (9)	0.9380 (17)	−0.0371 (9)	0.086 (5)	0.701 (5)
H6AA	0.0975	0.9781	−0.0788	0.103*	0.701 (5)
S1B	0.1113 (9)	0.9171 (14)	−0.0430 (7)	0.104 (4)	0.299 (5)
C6B	0.1633 (18)	0.8181 (18)	−0.0295 (10)	0.100 (11)	0.299 (5)
H6BA	0.1852	0.7779	−0.0676	0.120*	0.299 (5)
O1	0.11079 (10)	0.71831 (9)	0.21860 (10)	0.0469 (4)	
O2	0.21138 (11)	0.80238 (11)	0.27006 (12)	0.0647 (5)	
O3	−0.03265 (9)	0.82628 (10)	0.19487 (10)	0.0453 (4)	
O4	−0.00902 (9)	0.96587 (11)	0.23639 (12)	0.0595 (5)	
O5	−0.06263 (9)	0.61837 (11)	0.21667 (11)	0.0582 (5)	
H18	−0.1147	0.6295	0.2189	0.087*	
H19	−0.0547	0.5646	0.2351	0.087*	
N1	0.06793 (11)	0.61120 (12)	0.07465 (11)	0.0416 (4)	
N2	−0.05051 (12)	0.73028 (13)	0.04201 (13)	0.0491 (5)	
C1	0.01571 (12)	0.89209 (14)	0.20888 (13)	0.0376 (5)	
C2	0.14652 (14)	0.79424 (14)	0.23075 (14)	0.0406 (5)	
C3	0.10954 (12)	0.88032 (14)	0.19092 (13)	0.0375 (5)	
H3	0.1390	0.9340	0.2123	0.045*	
C4	0.12234 (12)	0.87804 (15)	0.10066 (14)	0.0426 (5)	
C5	0.09144 (18)	0.9444 (2)	0.04875 (19)	0.0722 (8)	
H5	0.0695	1.0000	0.0670	0.087*	
C7	0.16463 (17)	0.81237 (18)	0.05892 (17)	0.0561 (7)	
H7	0.1929	0.7608	0.0830	0.067*	
C8	0.12881 (14)	0.55364 (16)	0.09565 (16)	0.0515 (6)	
H8	0.1428	0.5489	0.1499	0.062*	
C9	0.17174 (16)	0.50113 (19)	0.04116 (19)	0.0639 (7)	
H9	0.2145	0.4623	0.0578	0.077*	
C10	0.14997 (19)	0.5074 (2)	−0.0387 (2)	0.0747 (9)	
H10	0.1778	0.4723	−0.0771	0.090*	
C11	0.08674 (18)	0.5659 (2)	−0.06165 (17)	0.0659 (7)	
H11	0.0713	0.5706	−0.1156	0.079*	
C12	0.04632 (14)	0.61766 (16)	−0.00342 (14)	0.0455 (6)	
C13	−0.02145 (15)	0.68297 (17)	−0.02135 (15)	0.0478 (6)	
C14	−0.05387 (19)	0.6973 (2)	−0.09774 (17)	0.0738 (9)	
H14	−0.0336	0.6638	−0.1413	0.089*	
C15	−0.1155 (2)	0.7605 (3)	−0.1090 (2)	0.0865 (10)	
H15	−0.1378	0.7699	−0.1601	0.104*	
C16	−0.1442 (2)	0.8096 (2)	−0.0452 (2)	0.0820 (10)	
H16	−0.1855	0.8539	−0.0520	0.098*	
C17	−0.11076 (18)	0.79227 (18)	0.0303 (2)	0.0716 (9)	
H17	−0.1310	0.8249	0.0744	0.086*	
O6	0.30803 (11)	0.96079 (13)	0.28178 (15)	0.0902 (7)	
H20	0.2790	0.9120	0.2835	0.135*	
H21	0.3574	0.9424	0.2919	0.135*	
O7	0.27699 (11)	0.62083 (13)	0.27671 (16)	0.0973 (8)	
H22	0.2451	0.6675	0.2774	0.146*	
H23	0.2433	0.5767	0.2851	0.146*	

### Atomic displacement parameters (Å^2^)

**Table:**

	*U*^11^	*U*^22^	*U*^33^	*U*^12^	*U*^13^	*U*^23^
Zn1	0.03884 (16)	0.03100 (15)	0.04402 (17)	−0.00086 (10)	−0.00143 (11)	−0.00347 (11)
S1A	0.0931 (16)	0.102 (2)	0.0563 (9)	−0.0365 (14)	0.0254 (9)	−0.0082 (10)
C6A	0.053 (5)	0.109 (11)	0.096 (9)	0.016 (5)	0.005 (5)	0.048 (7)
S1B	0.113 (6)	0.124 (8)	0.076 (4)	−0.004 (4)	0.021 (3)	0.031 (3)
C6B	0.078 (12)	0.041 (8)	0.18 (3)	−0.002 (8)	0.059 (12)	0.034 (10)
O1	0.0499 (9)	0.0316 (8)	0.0592 (10)	0.0009 (7)	−0.0149 (8)	0.0001 (7)
O2	0.0510 (10)	0.0531 (11)	0.0899 (14)	−0.0023 (8)	−0.0338 (10)	0.0044 (9)
O3	0.0337 (8)	0.0366 (9)	0.0655 (11)	−0.0017 (7)	0.0039 (8)	−0.0123 (8)
O4	0.0452 (9)	0.0363 (9)	0.0968 (14)	0.0014 (7)	0.0116 (9)	−0.0224 (9)
O5	0.0435 (9)	0.0411 (9)	0.0899 (13)	0.0012 (7)	0.0131 (9)	0.0187 (9)
N1	0.0401 (10)	0.0402 (10)	0.0446 (11)	−0.0004 (8)	0.0010 (8)	−0.0022 (9)
N2	0.0494 (12)	0.0404 (11)	0.0574 (13)	0.0018 (9)	−0.0142 (10)	0.0001 (10)
C1	0.0366 (11)	0.0317 (11)	0.0444 (13)	0.0000 (9)	0.0015 (9)	−0.0017 (10)
C2	0.0382 (12)	0.0396 (12)	0.0442 (13)	0.0046 (10)	−0.0028 (10)	−0.0005 (10)
C3	0.0322 (11)	0.0307 (11)	0.0497 (13)	−0.0019 (9)	−0.0042 (10)	−0.0026 (10)
C4	0.0318 (11)	0.0440 (13)	0.0518 (14)	−0.0058 (9)	0.0033 (10)	0.0081 (11)
C5	0.0660 (17)	0.077 (2)	0.073 (2)	0.0156 (15)	0.0104 (15)	0.0262 (16)
C7	0.0586 (16)	0.0536 (16)	0.0560 (17)	−0.0071 (12)	0.0175 (13)	−0.0004 (13)
C8	0.0479 (13)	0.0487 (14)	0.0579 (16)	0.0061 (11)	−0.0070 (12)	−0.0047 (12)
C9	0.0522 (15)	0.0572 (17)	0.082 (2)	0.0089 (12)	0.0002 (15)	−0.0139 (15)
C10	0.0691 (19)	0.080 (2)	0.075 (2)	0.0102 (16)	0.0159 (17)	−0.0264 (17)
C11	0.0748 (18)	0.078 (2)	0.0450 (15)	−0.0004 (16)	0.0044 (13)	−0.0155 (14)
C12	0.0455 (13)	0.0467 (14)	0.0441 (14)	−0.0095 (11)	0.0024 (11)	−0.0032 (11)
C13	0.0495 (13)	0.0500 (15)	0.0439 (14)	−0.0112 (11)	−0.0053 (11)	0.0067 (11)
C14	0.0690 (19)	0.104 (2)	0.0487 (17)	−0.0068 (17)	−0.0068 (15)	0.0143 (16)
C15	0.081 (2)	0.101 (3)	0.077 (2)	−0.011 (2)	−0.0295 (19)	0.034 (2)
C16	0.070 (2)	0.063 (2)	0.113 (3)	0.0004 (15)	−0.042 (2)	0.0220 (19)
C17	0.0668 (18)	0.0556 (17)	0.092 (2)	0.0114 (14)	−0.0271 (17)	−0.0064 (15)
O6	0.0429 (11)	0.0615 (12)	0.166 (2)	0.0055 (9)	−0.0057 (12)	−0.0011 (13)
O7	0.0408 (10)	0.0598 (13)	0.191 (2)	0.0105 (9)	−0.0077 (13)	0.0120 (14)

### Geometric parameters (Å, °)

**Table:**

Zn1—O1	2.0001 (15)	C3—H3	0.9800
Zn1—O3	2.0224 (15)	C4—C7	1.361 (3)
Zn1—O5	1.9596 (15)	C4—C5	1.386 (3)
Zn1—N1	2.1123 (18)	C5—H5	0.9317
Zn1—N2	2.100 (2)	C7—H7	0.9630
S1A—C7	1.672 (3)	C8—C9	1.369 (3)
S1A—C6A	1.79 (2)	C8—H8	0.9300
C6A—C5	1.461 (16)	C9—C10	1.373 (4)
C6A—H6AA	0.9300	C9—H9	0.9300
S1B—C6B	1.68 (3)	C10—C11	1.376 (4)
S1B—C5	1.606 (14)	C10—H10	0.9300
C6B—C7	1.469 (17)	C11—C12	1.385 (3)
C6B—H6BA	0.9300	C11—H11	0.9300
O1—C2	1.261 (2)	C12—C13	1.473 (3)
O2—C2	1.231 (3)	C13—C14	1.385 (3)
O3—C1	1.254 (2)	C14—C15	1.361 (4)
O4—C1	1.233 (2)	C14—H14	0.9300
O5—H18	0.8499	C15—C16	1.358 (5)
O5—H19	0.8500	C15—H15	0.9300
N1—C8	1.331 (3)	C16—C17	1.385 (4)
N1—C12	1.344 (3)	C16—H16	0.9300
N2—C17	1.335 (3)	C17—H17	0.9300
N2—C13	1.340 (3)	O6—H20	0.8499
C1—C3	1.540 (3)	O6—H21	0.8501
C2—C3	1.536 (3)	O7—H22	0.8500
C3—C4	1.512 (3)	O7—H23	0.8501
			
O5—Zn1—O1	104.60 (7)	C4—C5—C6A	119.0 (9)
O5—Zn1—O3	101.65 (6)	C4—C5—S1B	110.2 (6)
O1—Zn1—O3	88.61 (6)	C6A—C5—S1B	10.7 (17)
O5—Zn1—N2	110.18 (7)	C4—C5—H5	122.6
O1—Zn1—N2	144.76 (7)	C6A—C5—H5	116.9
O3—Zn1—N2	89.78 (7)	S1B—C5—H5	126.7
O5—Zn1—N1	101.30 (7)	C4—C7—S1A	113.3 (2)
O1—Zn1—N1	90.98 (7)	C4—C7—C6B	117.4 (11)
O3—Zn1—N1	156.38 (7)	S1A—C7—C6B	7.3 (11)
N2—Zn1—N1	77.11 (7)	C4—C7—H7	124.8
C7—S1A—C6A	95.9 (5)	S1A—C7—H7	121.8
C5—C6A—S1A	101.1 (10)	C6B—C7—H7	117.7
C5—C6A—H6AA	129.4	N1—C8—C9	123.1 (2)
S1A—C6A—H6AA	129.4	N1—C8—H8	118.5
C6B—S1B—C5	100.6 (10)	C9—C8—H8	118.5
S1B—C6B—C7	100.9 (15)	C8—C9—C10	118.2 (3)
S1B—C6B—H6BA	129.5	C8—C9—H9	120.9
C7—C6B—H6BA	129.5	C10—C9—H9	120.9
C2—O1—Zn1	124.22 (14)	C9—C10—C11	119.7 (3)
C1—O3—Zn1	123.42 (13)	C9—C10—H10	120.2
Zn1—O5—H18	117.2	C11—C10—H10	120.2
Zn1—O5—H19	133.4	C10—C11—C12	119.1 (3)
H18—O5—H19	107.9	C10—C11—H11	120.4
C8—N1—C12	119.0 (2)	C12—C11—H11	120.4
C8—N1—Zn1	125.22 (16)	N1—C12—C11	120.9 (2)
C12—N1—Zn1	115.63 (15)	N1—C12—C13	115.4 (2)
C17—N2—C13	119.0 (2)	C11—C12—C13	123.7 (2)
C17—N2—Zn1	124.7 (2)	N2—C13—C14	120.7 (2)
C13—N2—Zn1	116.26 (16)	N2—C13—C12	115.4 (2)
O4—C1—O3	122.49 (19)	C14—C13—C12	123.9 (3)
O4—C1—C3	118.79 (18)	C15—C14—C13	119.9 (3)
O3—C1—C3	118.73 (18)	C15—C14—H14	120.0
O2—C2—O1	123.4 (2)	C13—C14—H14	120.0
O2—C2—C3	118.29 (19)	C16—C15—C14	119.6 (3)
O1—C2—C3	118.20 (19)	C16—C15—H15	120.2
C4—C3—C2	110.86 (17)	C14—C15—H15	120.2
C4—C3—C1	109.04 (17)	C15—C16—C17	118.6 (3)
C2—C3—C1	112.53 (17)	C15—C16—H16	120.7
C4—C3—H3	108.1	C17—C16—H16	120.7
C2—C3—H3	108.1	N2—C17—C16	122.2 (3)
C1—C3—H3	108.1	N2—C17—H17	118.9
C7—C4—C5	110.6 (2)	C16—C17—H17	118.9
C7—C4—C3	125.9 (2)	H20—O6—H21	103.8
C5—C4—C3	123.5 (2)	H22—O7—H23	102.8
			
C7—S1A—C6A—C5	−2.2 (10)	C7—C4—C5—C6A	−3.1 (10)
C5—S1B—C6B—C7	−1.9 (17)	C3—C4—C5—C6A	177.0 (9)
O5—Zn1—O1—C2	−136.45 (18)	C7—C4—C5—S1B	3.7 (7)
O3—Zn1—O1—C2	−34.78 (18)	C3—C4—C5—S1B	−176.2 (7)
N2—Zn1—O1—C2	52.9 (2)	S1A—C6A—C5—C4	3.4 (13)
N1—Zn1—O1—C2	121.60 (18)	S1A—C6A—C5—S1B	−33 (5)
O5—Zn1—O3—C1	139.19 (18)	C6B—S1B—C5—C4	−0.9 (14)
O1—Zn1—O3—C1	34.56 (18)	C6B—S1B—C5—C6A	145 (6)
N2—Zn1—O3—C1	−110.24 (19)	C5—C4—C7—S1A	1.1 (3)
N1—Zn1—O3—C1	−54.7 (3)	C3—C4—C7—S1A	−179.0 (2)
O5—Zn1—N1—C8	−72.87 (18)	C5—C4—C7—C6B	−5.5 (12)
O1—Zn1—N1—C8	32.23 (18)	C3—C4—C7—C6B	174.4 (12)
O3—Zn1—N1—C8	121.0 (2)	C6A—S1A—C7—C4	0.8 (7)
N2—Zn1—N1—C8	178.76 (19)	C6A—S1A—C7—C6B	127 (10)
O5—Zn1—N1—C12	111.89 (16)	S1B—C6B—C7—C4	4.5 (18)
O1—Zn1—N1—C12	−143.01 (15)	S1B—C6B—C7—S1A	−52 (9)
O3—Zn1—N1—C12	−54.3 (2)	C12—N1—C8—C9	1.0 (3)
N2—Zn1—N1—C12	3.51 (15)	Zn1—N1—C8—C9	−174.14 (18)
O5—Zn1—N2—C17	82.5 (2)	N1—C8—C9—C10	−0.9 (4)
O1—Zn1—N2—C17	−107.2 (2)	C8—C9—C10—C11	0.4 (4)
O3—Zn1—N2—C17	−19.9 (2)	C9—C10—C11—C12	0.2 (4)
N1—Zn1—N2—C17	179.9 (2)	C8—N1—C12—C11	−0.4 (3)
O5—Zn1—N2—C13	−99.53 (17)	Zn1—N1—C12—C11	175.18 (18)
O1—Zn1—N2—C13	70.9 (2)	C8—N1—C12—C13	−179.9 (2)
O3—Zn1—N2—C13	158.13 (17)	Zn1—N1—C12—C13	−4.4 (2)
N1—Zn1—N2—C13	−2.06 (16)	C10—C11—C12—N1	−0.2 (4)
Zn1—O3—C1—O4	−177.78 (18)	C10—C11—C12—C13	179.4 (2)
Zn1—O3—C1—C3	2.3 (3)	C17—N2—C13—C14	−0.7 (4)
Zn1—O1—C2—O2	−178.94 (18)	Zn1—N2—C13—C14	−178.78 (19)
Zn1—O1—C2—C3	−2.1 (3)	C17—N2—C13—C12	178.6 (2)
O2—C2—C3—C4	107.2 (2)	Zn1—N2—C13—C12	0.5 (3)
O1—C2—C3—C4	−69.8 (2)	N1—C12—C13—N2	2.6 (3)
O2—C2—C3—C1	−130.3 (2)	C11—C12—C13—N2	−176.9 (2)
O1—C2—C3—C1	52.6 (3)	N1—C12—C13—C14	−178.2 (2)
O4—C1—C3—C4	−109.2 (2)	C11—C12—C13—C14	2.3 (4)
O3—C1—C3—C4	70.7 (2)	N2—C13—C14—C15	0.5 (4)
O4—C1—C3—C2	127.4 (2)	C12—C13—C14—C15	−178.7 (3)
O3—C1—C3—C2	−52.7 (3)	C13—C14—C15—C16	0.6 (5)
C2—C3—C4—C7	−3.5 (3)	C14—C15—C16—C17	−1.4 (5)
C1—C3—C4—C7	−127.9 (2)	C13—N2—C17—C16	−0.2 (4)
C2—C3—C4—C5	176.4 (2)	Zn1—N2—C17—C16	177.8 (2)
C1—C3—C4—C5	52.0 (3)	C15—C16—C17—N2	1.2 (5)

### Hydrogen-bond geometry (Å, °)

**Table:**

*D*—H···*A*	*D*—H	H···*A*	*D*···*A*	*D*—H···*A*
O5—H18···O7^i^	0.85	1.74	2.568 (2)	165
O5—H19···O4^ii^	0.85	1.83	2.618 (2)	155
O6—H20···O2	0.85	1.94	2.784 (2)	172
O6—H21···O4^iii^	0.85	2.22	2.943 (2)	144
O6—H21···O3^iii^	0.85	2.45	3.238 (2)	154
O7—H22···O2	0.85	2.04	2.847 (3)	158
O7—H22···O1	0.85	2.47	3.165 (2)	139
O7—H23···O6^iv^	0.85	1.88	2.700 (3)	162

Symmetry codes: (i) *x*−1/2, *y*, −*z*+1/2; (ii) −*x*, *y*−1/2, −*z*+1/2; (iii) *x*+1/2, *y*, −*z*+1/2; (iv) −*x*+1/2, *y*−1/2, *z*.
